# Improving Lipid Content in the Diatom *Phaeodactylum tricornutum* by the Knockdown of the Enoyl-CoA Hydratase Using CRISPR Interference

**DOI:** 10.3390/cimb46100649

**Published:** 2024-09-28

**Authors:** Wenfeng Guo, Yuwei Weng, Wenkai Ma, Chaofeng Chang, Yuqing Gao, Xuguang Huang, Feng Zhang

**Affiliations:** 1 College of Biological Sciences and Technology, Minnan Normal University, Zhangzhou 363000, China; 2 School of Advanced Manufacturing, Fuzhou University, Quanzhou 362251, China; 3 College of Chemistry and Environmental Science, Minnan Normal University, Zhangzhou 363000, China

**Keywords:** *Phaeodactylum tricornutum*, lipid, CRIRSPi, enoyl-CoA hydratase

## Abstract

The diatom *Phaeodactylum tricornutum* shows potential as a source for biofuel production because of its considerable lipid content. Fatty acid β-oxidation plays a critical role in lipid breakdown. However, we still have a limited understanding of the role of fatty acid β-oxidation in lipid content in this microalga. In our study, we utilized a CRISPR interference method to reduce the expression of enoyl-CoA hydratase (PtECH), which is involved in the hydration of trans-2-enoyl-CoA to produce 3-hydroxyacyl-CoA during the β-oxidation pathway. Using this method, we developed two transgenic lines, PtECH21 and PtECH1487, which resulted from interference at two different sites of the *PtECH* gene, respectively. RT-qPCR analysis confirmed that the mRNA levels of PtECH in both mutants were significantly lower compared to the wild type. Surprisingly, the lipid content of both mutants increased notably. Additionally, both knockdown mutants exhibited higher chlorophyll content and improved photosynthetic efficiency of the photosystem II compared to the wild type. This study introduces a new approach for enhancing lipid content in *P. tricornutum* and expands our knowledge of the functions of enoyl-CoA hydratase in microalgae.

## 1. Introduction

Diatoms have garnered significant attention as a potential source of lipids with industrial applications. For instance, diatoms like *Phaeodactylum tricornutum* and *Nannochloropsis* sp. can produce ω-3 polyunsaturated fatty acids (ω-3 PUFAs), particularly eicosapentaenoic acid (EPA). Many ω-3 PUFAs are known for their various health benefits, including anti-tumor, anti-inflammatory, and cardiovascular disease prevention properties [1]. Some diatoms can synthesize approximately 20% of EPA, highlighting their potential value in the food and feed industry [2]. Moreover, diatoms contain various bioactive lipids and their derivatives, including steroids and carotenoids, which have been found to possess cytotoxic, anti-cancer, anti-inflammatory, and antimycobacterial activities [3,4,5]. With the decrease in petroleum reserves, biofuels have become an attractive option due to their renewable nature and recyclability. Diatoms exhibit a significant capacity for the biosynthesis of triacylglycerols (TAGs), which make up more than 45% of their weight in certain of them, thus making them important sources for biofuel production [6].

Over the past few decades, genetic engineering strategies have been widely used to improve the lipid content of diatoms [7,8]. As a model diatom, *P. tricornutum* is the most commonly used for genetic modification. Up to now, three primary genetic techniques have been employed to enhance lipid content in *P. tricornutum*: gene overexpression [9,10,11,12,13], RNA interference (RNAi) [14,15,16,17,18], and gene editing [19,20,21,22]. However, CRISPR interference (CRISPRi) technology has not yet been applied to *P. tricornutum*.

CRISPRi, derived from the CRISPR (clustered regularly interspaced palindromic repeats) system, represses gene transcription using a catalytically inactive version of Cas9 called dead Cas9 (dCas9). The dCas9 contains two-point mutations in both its endonuclease domains, RuvC (D10A) and HNH (H840A) [23]. As a result, when combined with single guide RNA (sgRNA), dCas9 can bind to the target gene and stop transcription initiation or elongation. CRISPRi technology has been employed to enhance metabolite productivity in several microalgae species, including *Chlamydomonas reinhardtii* [24], *Synechcocystis* sp. PCC 6803 [25], *S. elongatus* PCC 7942 [26], and *Synechcocystis* sp. PCC 7002 [27]. With the help of CRISPRi technology, an engineered *Synechocystis* sp. strain increased lactate production by 2-fold compared to the original cell lines [27].

Regardless of genetic techniques, previous studies on enhancing lipid content in *P. tricornutum* can be classified into two strategies: (1) optimizing the lipid biosynthetic pathway and (2) inhibiting the lipid catabolic pathway [7,28]. However, most previous studies focused on strategy 1 [29]. Fatty acid β-oxidation is a significant pathway for the degradation of most lipids in eukaryotes. It lies in the conversion of fatty acid into acetyl-CoAs through successive β-oxidation spirals [30]. Each cycle relies on at least four significant enzymes: acyl-CoA dehydrogenase (EC 1.3.8.1), enoyl-CoA hydratase (ECH) (EC 4.2.1.150), L-3-hydroxyacyl CoA dehydrogenase (EC 1.1.1.35), and β-ketothiolase (EC 2.3.1.16) [31]. Among them, ECH catalyzes the hydration of the double bond between C-2 and C-3 on acyl-CoA [32]. In 2015, Feng and colleagues demonstrated that downregulating ECH (Phatr3_J55192) could reduce fatty acid catabolism but promote lipid accumulation [33]. In this study, we inhibited lipid catabolism by disturbing the expression of enoyl-CoA hydratase in *P. tricornutum* (PtECH) using the CRISPRi approach. As a result, the PtECH knockdown mutant showed an enhanced lipid content compared to the wild type. This study strongly suggests that the CRISPRi-mediated knockdown of target genes effectively enhances lipid content in *P. tricornutum*.

## 2. Materials and Methods

### 2.1. Microbial Strains and Culture Conditions

*Escherichia coli* DH5α was cultured in a Luria broth or on Luria agar. *E. coli* DH10B strains containing plasmids pTA-MOB were grown in Luria broth or on agar supplemented with gentamicin (50 mg/L) [34]. The diatom *Phaeodactylum tricornutum* Bohlin, obtained from the State Key Laboratory for Marine Environmental Science of Xiamen University, was grown in an L1 medium (Cellbio, Xiamen, China) [35] without silica at 22 °C under cool white fluorescent lights (140 μmol photons m^−2^s^−1^) with a photoperiod of 16 h of light and 8 h of darkness. To prevent bacterial contamination, 50 µg/mL of kanamycin was added to the L1 medium. For growth on solid media, the algae were cultured on 1/2 × L1 medium containing 1% agar.

### 2.2. Plasmid Construction

To create the inactive Cas9 vector pPtGE35-dCas9, a PCR-based site-directed mutagenesis approach was used with the plasmid diaTevCas9 (Catalog number 107999, Addgene, Watertown, MA, USA) as a template following standard protocols [36]. To build two CRISPRi constructs, two sets of oligonucleotides were designed using a custom Python script to search for TevCas9 gRNA target sites in the PtECH gene. These oligonucleotide pairs were denatured for 3 min at 94 °C, annealed for 30 min at room temperature, and then diluted 100 times. After *Bsa*I digestion, pPtGE35-dCas9 was ligated with the annealed oligonucleotides using a T4 ligase kit following the manufacturer’s instructions (New England Biolabs, Ipswich, UK). All the constructs were further confirmed by Sanger sequencing (Azenta Life Science, Suzhou, China).

### 2.3. Generation of Transgenic P. tricornutum by Bacteria Conjugation

The conjugation protocols were carried out according to the methods described by Karas and colleagues with some modifications [37]. Briefly, 200 μL of *P. tricornutum* (10^8^/mL) was combined with 200 μL of *E. coli* cells. The cell mixture was then spread on a 1/2 × L1 agar plate supplemented with 5% LB and incubated at 30 °C in the dark for 90 min. The cultures were then transferred to 22 °C in the light and grown for 2 days. Afterward, 1 mL of L1 media was added to the plate, and the cells were scraped. A total of 400 μL of cells were plated on a 1/2 × L1 agar plate supplemented with 50 μg/mL of the antibiotic zeocin (InvivoGen, San Diego, CA, USA). Following a 21-day incubation at 22 °C with light, six colonies were randomly selected and inoculated into a 6-well plate including 5 mL of L1 media with 50 µg/mL of the antibiotic zeocin (InvivoGen, San Diego, CA, USA). The plate was placed in an incubator at 22 °C under light. After 7 days of growth, 1 mL of the cultures was transferred to a clean tube and pelleted at 3000× *g* for 10 min. The collected cells were then resuspended in 50 μL Tris-EDTA buffer consisting of 10 mM Tris-HCl (pH 8.0) and 1 mM EDTANa_2_, followed by lysis at 65 °C for 30 min. In total, 2 μL of cell lysates were used as the template for PCR verification. The PCR primers spanned the annealed oligonucleotides in the CRISPRi vector. After the PCR products were analyzed via electrophoresis, they were sequenced at GENEWIZ (Azenta Life Science, China). All the primers are listed in Supplementary Table S1.

### 2.4. RNA Isolation and Quantitative PCR Experiments

To extract total RNA from *P. tricornutum*, 1 L of the cell cultures was spun for 15 min at 3000× *g* at 4 °C. The cell pellets were frozen at −80° C overnight and then subjected to freeze-drying with a vacuum freeze dryer. The freeze-dried pellets were ground in liquid nitrogen, and total RNA was extracted using TRIzol reagent (Lablead, Beijing, China) following the manufacturer’s protocols. For quantitative PCR experiments, 1 μg of total RNA was reverse transcribed into cDNA using a cDNA reverse transcription kit (Lablead, Beijing, China) per the manufacturer’s instructions. A quantitative PCR analysis was conducted using SYBR Green PCR Master Mix (Lablead, Beijing, China) and a Quant Studio 6/7 Flex System (Applied Biosystems, Waltham, MA, USA). The histone H4 gene was used as the internal reference gene for normalizing the expression of the target gene. The relative expression of the target gene was estimated using the 2^−∆∆Ct^ method [38]. The specific primers used in quantitative PCR are detailed in Supplementary Table S1.

### 2.5. Analysis of Growth Rates and Photosynthesis

In order to analyze the growth of the wild-type and knockdown mutants, 10^6^ algae cells were inoculated in 100 mL of L1 media and cultivated for 14 days under the conditions mentioned in Section 2.1. The density of cell cultures was measured every other day using an Accuri™ C6 Plus flow cytometry (BD, San Jose, CA, USA). Additionally, for photosynthesis assessment, the chlorophyll content and the chlorophyll fluorescence parameter Fv/Fm of the wild-type and knockdown mutants were measured on the seventh day using a Phyto-PAM fluorometry equipped with a System I emitter-detector unit (Walz, Effeltrich, Germany). Aliquots (2 mL) of algal cultures were dark-adapted for 10 min before measuring chlorophyll content and Fv/Fm. The “Meas.Freq.” was set to 32 for measuring chlorophyll content and 2 for measuring Fv/Fm. All the other parameters were set to default as per the manufacturer’s instructions.

### 2.6. Measurement of TAG Content and Fatty Acid Composition

To analyze the TAG content, 3 mL of 7-day incubation cultures were mixed with 3 μL of Nile red solution (250 μg/mL in acetone). After keeping the mixture at 37 °C for 30 min, the fluorescence of the mixture was excited at 530 nm and the fluorescent emission was measured at 585 nm using a Tecan Fluorescence Spectrophotometer (Tecan, Männedorf, Switzerland). To analyze the fatty acid composition, the total lipids were extracted from 100 mL cultures in the stationary growth phase following the method described by Axelsson and Gentili [39]. The algal paste was mixed with 8 mL of a 2:1 chloroform/methanol (*v*/*v*) mixture using a vortex. Then, 2 mL of a 0.73% NaCl solution was added to the mixture and swirled. After 5 min of centrifugation at 13,000× *g*, the organic layer was collected and dried with nitrogen. The fatty acid residues from lipids were transformed to methyl esters in 1 mL of 5.3% H_2_SO_4_ in pure CH_3_OH at 100 °C. After 1 h of incubation, the fatty acid methyl esters were extracted using the method described by Hao et al. [19]. Finally, the fatty acid composition was determined using a 7890 B gas chromatography (Agilent, Santa Clara, CA, USA) equipped with a flame ionization detector and an SH-2560 capillary column (100 m × 0.25 mm × 0.20 μm) (SHIMADZU, Kyoto, Japan). Nitrogen was employed as the carrier gas at a constant flow rate of 1.5 mL/min. The method parameters were as follows: injection volume 1 μL; a split ratio of 10:1; the temperatures of the injection port and the detector were both 250 °C; and oven temperature program with starting temperature 140 °C, hold for 1 min, then increase at a rate of 4 °C/min to 240 °C, and hold for 5 min.

### 2.7. Phylogenetic Analysis

The protein sequence of enoyl-CoA hydratase (PtECH) from *P. tricornutum* was used as a query (XP_002185363.1) to find similar sequences from the non-redundant protein sequence databases in the National Center for Biotechnology Information using BLAST [40]. This search found 30 entries with high amino acid sequence homology to the PtECH protein. A phylogenetic tree was yielded using the neighbor-joining method [41] in the MEGA 11 software [42] based on the ClustalW alignment comparison [43]. The bootstrap is set to 1000 replications [44], and all the other parameters are at their default settings. The enoyl-CoA hydratase protein domain was identified using the SMART 9 tool (http://smart.embl-heidelberg.de/smart/set_mode.cgi?NORMAL=1 (accessed on 24 September 2024)) [45] and visualized using the IBS 2.0 software [46].

### 2.8. Statistical Analysis

Data for this study were shown as means and standard deviations from three replicates. D’Agostino–Pearson test was used to assess the normality of data distribution, and Tukey’s multiple comparisons were used to test multiple comparisons. The statistical analyses of the data were performed with a one-way analysis of variance, and *p* < 0.05 was considered statistically significant.

## 3. Results

### 3.1. Sequence Analysis of PtECH Protein

The PtECH protein comprised 269 amino acids and contained an enoyl-CoA hydratase domain (Supplementary Figure S1a). Similar to PtECH, the other orthologs also contained the enoyl-CoA hydratase domain, indicating its conservation in eukaryotes (Supplementary Figure S1a). Through BLAST research, 35 entries with high amino acid sequence homology to the PtECH protein were selected from the NCBI database. Sequence alignment and phylogenetics revealed that ECH homologs from 35 species clustered into three groups (Supplementary Figure S1b). In the phylogenetic tree, PtECH was closely related to diatoms, with all the diatoms separated from the other two groups comprising bacteria and fungi. These results suggest that diatom ECHs are well conserved and may have similar biological functions.

### 3.2. Generation of PtECH Knockdown P. tricornutum

**Figure 1:**
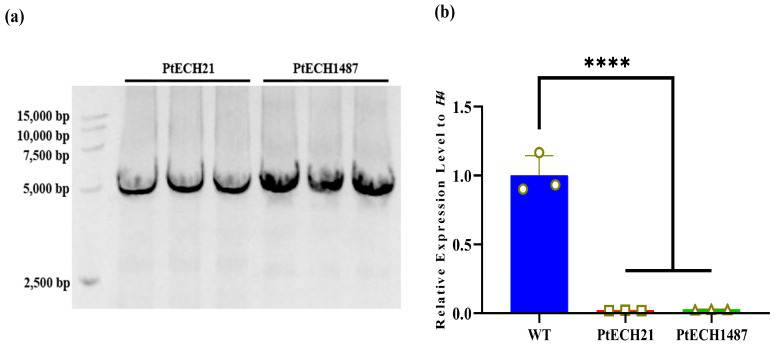
Generation of *PtECH* knockdown *P. tricornutum*. (**a**) Transformation of CRISPRi vector into *P. tricornutum* verified by PCR. (**b**) Expression of *PtECH* transcripts measured by qPCR. Each value represents mean ± SD (**** *p* ≤ 0.0001). PtECH21 and PtECH1487: two transgenic microalgae; WT: wild-type microalgae.

To create PtECH knockdown mutants using the CRISPRi technique, we introduced two different CRISPRi constructs into *P. tricornutum* by conjugating with *E. coli*. To confirm the integration of the constructs into *P. tricornutum*, we amplified and sequenced a region of the constructs containing pairs of oligonucleotides. As shown in Figure 1a, both transgenic lines (PtECH21 and PtECH1487) displayed the expected amplicon, indicating the successful introduction of the CRISPRi constructs. To assess the impact of the CRISPRi constructs on PtECH mRNA abundance in *P. tricornutum*, we selected wild-type cells and two transgenic lines for an RT-qPCR analysis. As illustrated in Figure 1b, the PtECH transcript levels exhibited a significant reduction of 98% and 97% in PtECH21 and PtECH1487 mutants, respectively. These results demonstrate that the expression of the *PtECH* gene in *P. tricornutum* can be suppressed using the CRISPRi strategy.

### 3.3. Effects of the PtECH Knockdown on Lipid Contents

**Figure 2:**
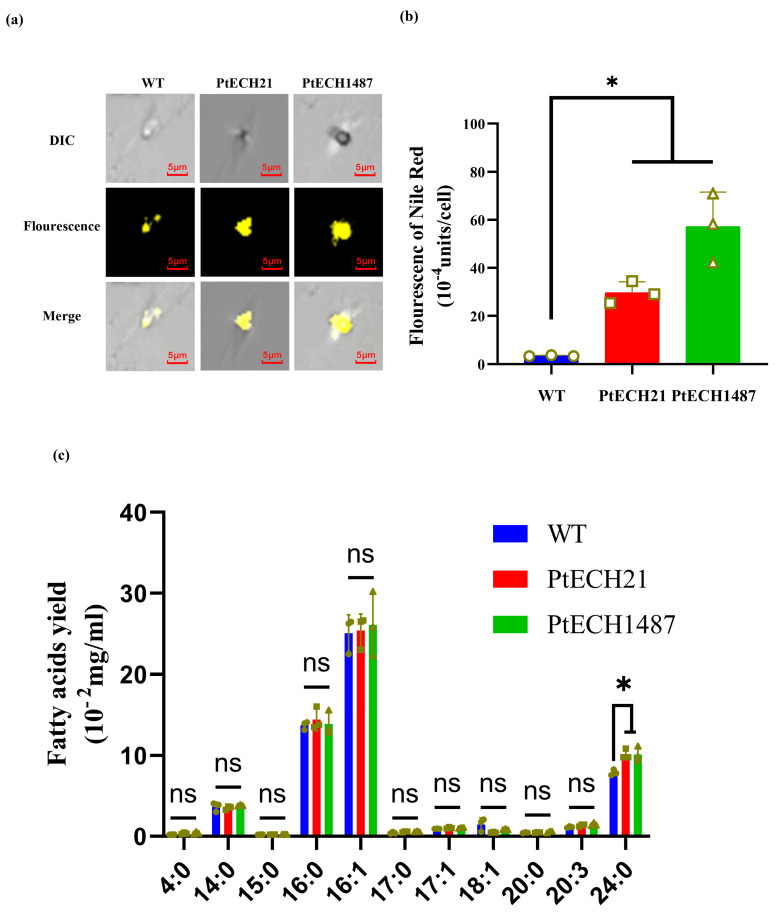
Effects of the *PtECH* knockdown on lipid content. (**a**) The confocal images of Nile red-stained algal cells. DIC, differential interference contrast. Fluorescence, yellow fluorescence of oil bodies. (**b**) The accumulation of the total lipid was determined by a Nile red assay. (**c**) The yield of different fatty acids produced by microalgae. Each value represents mean ± SD (* *p* ≤ 0.05). PtECH21 and PtECH1487: two transgenic microalgae; WT: wild-type microalgae; ns: not significant.

Enoyl-CoA hydratase (ECH) is responsible for the second step of the β-oxidation pathway, which breaks down fatty acids. The results, depicted in Figure 2a, show significantly higher fluorescence signals from oil bodies in the mutant cells compared to the wild type. Notably, fluorescence intensity analysis revealed that the neutral lipid content per cell was 7-fold higher in the PtECH21 mutants and 20-fold higher in the PtECH1487 mutants compared to the wild type (Figure 2b). Additionally, we analyzed the fatty acid composition of *P. tricornutum* at the stationary growth phase. As illustrated in Figure 2c, palmitic acid (16:0) and palmitoleic acid (16:1) were the primary fatty acids in both the wild-type and knockdown strains. Although most fatty acids in the knockdown lines were similar to those in the wild-type lines, the PtECH knockdown lines showed an increase in C24 fatty acids. These findings collectively indicate that reducing PtECH expression is an effective method for boosting lipid accumulation.

### 3.4. Impacts of the PtECH Knockdown on Growth and Photosynthesis

**Figure 3:**
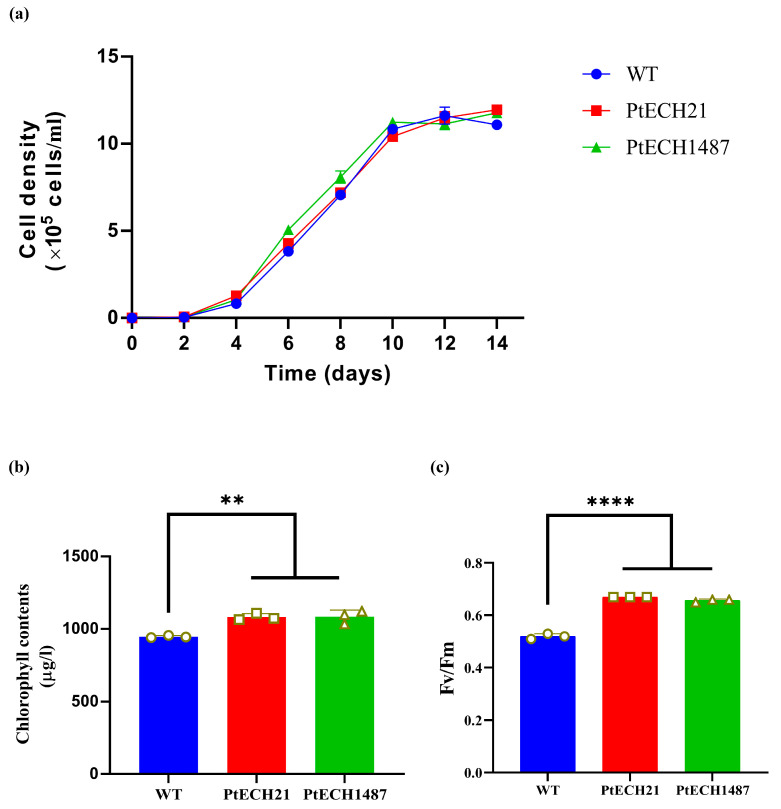
Impacts of the *PtECH* knockdown on the growth rates and chlorophyll parameters. (**a**) The growth curve of the microalgae. (**b**) The chlorophyll contents of the microalgae analyzed by Phyto-PAM fluorometry on day 5. (**c**) The chlorophyll fluorescence parameter Fm/Fv of the microalgae. Each value represents mean ± SD (** *p* ≤ 0.01; **** *p* ≤ 0.0001). PtECH21 and PtECH1487: two transgenic microalgae; WT: wild-type microalgae.

Next, we investigated the impact of reducing PtECH expression on the growth of *P. tricornutum* due to its crucial role in fatty acid breakdown. Figure 3a shows no significant difference in cell density between the knockdown lines and the wild type from day 1 to 14. However, we observed higher chlorophyll contents at day 5 in PtECH21 and PtECH1487 lines compared to the wild-type cells (Figure 3b). Since *P. tricornutum* relies on chlorophyll for organic matter synthesis, we proposed that PtECH may play a significant role in photosynthesis in this organism. To explore this further, we measured the chlorophyll fluorescence parameter Fv/Fm, which reflects the maximum quantum efficiency of PSII. As shown in Figure 3c, both knockdown lines exhibited a significant increase in the value of Fv/Fm compared to the wild type, suggesting that the knockdown of PtECH influenced photosynthesis in *P. tricornutum*.

## 4. Discussion

Many genetic engineering approaches have been used to boost lipid content in *P. tricornutum* [47]. In this study, we established a CRISPRi approach to increase lipid accumulation in *P. tricornutum* by inhibiting *PtECH* transcription. Similar to CRISPRi, RNAi can also be used to suppress gene expression. However, CRISPRi enables the genome-wide repression of gene expression, while RNAi operates at the mRNA level. Furthermore, the RNAi method relies entirely on the host RNAi pathway, which needs to be fully understood in diatoms, making the RNAi approach less predictable and specific [48]. Unlike CRISPR/Cas9 technology, CRISPRi disrupts gene expression without modifying the target DNA sequence. This indicates that CRISPRi-mediated gene silencing is inducible and reversible [49]. At this point, the CRISPRi method seems more meaningful than the CRISPR/Cas9 technique for investigating essential genes.

In theory, fatty acid β-oxidation is extremely important for most organisms, providing them with energy and carbon skeletons. However, the function of fatty acid β-oxidation enzymes in microalgae has been waiting for genetic validation. Recently, an acyl-CoA dehydrogenase (PtMACAD1) that catalyzes the initial step in the β-oxidation pathway was functionally described through reverse genetics [50]. Consistent with our observations, the *PtMACAD1* knockout strains showed increased lipid accumulation compared to the wild type [50]. These findings strengthened the possibility that the inactivity of fatty acid β-oxidation enzymes improved lipid accumulation. Although lipases are not involved in fatty acid β-oxidation, these enzymes also play a significant role in lipid breakdown. In *P. tricornutum*, OmTGL and Tgl1 lipases have been experimentally characterized [15,17]. In line with our findings, the knockdown of OmTGL or Tgl1 increased lipid accumulation in *P. tricornutum*. These data reinforce that inhibiting lipid degradation helps enhance lipid accumulation in *P. tricornutum*.

Chlorophyll, closely related to photosynthesis, is a lipid derivative surrounded by plastid membranes. Thus, lipid metabolism is associated with chlorophyll content. In our study, we observed that the knockdown of the *PtECH* gene led to the elevated chlorophyll content and photosynthesis efficiency of the photosystem II compared with the wild type. However, the knockout of acyl-CoA dehydrogenase (PtMACAD1), which catalyzes the dehydrogenation of acyl-CoA thioesters to the corresponding trans-2-enoyl-CoA in fatty acid β-oxidation, had no effect on chlorophyll content [50]. Similarly, when acyl-CoA thioesterase (ptTES1), which catalyzes the hydrolysis of acyl-CoA thioesters to free fatty acids and coenzyme A, was inactivated, it exhibited a photosynthetic performance similar to the wild type [19]. In *P. tricornutum*, many enzymes involved in fatty acid β-oxidation have other isozymes, suggesting they may have different functions. For example, *P. tricornutum* has three mitochondrial acyl-CoA dehydrogenases, i.e., PtMACAD1, ACD1, and ACD2, but only PtMACAD1 is directly involved in 16:0-CoA oxidation in vivo [50]. Also, three enoyl-CoA hydratases, Phatrdraft3_J54494, Phatrdraft3_J55192, and Phatrdraft3_J35240, were predicted to be located in the mitochondria of *P. tricornutum*. However, further exploration is needed to understand their biological function. On the other hand, enoyl-CoA hydratase plays a significant role in fatty acid β-oxidation, providing microalgae with considerable energy for survival. One possible explanation is that *PtECH* knockdown mutants require more energy from photosynthesis due to the decrease in fatty acid β-oxidation. Regardless, we need to examine this possibility in the future.

As mentioned above, diatoms can accumulate a greater quantity of lipids. In addition, they exhibit excellent qualities such as high growth rates, low nutritional requirements, and performance in large-scale cultures. Therefore, diatoms are considered to be more efficient and environmentally friendly biofuel production systems than most organisms [51]. Herein, we have developed a new methodology for enhancing diatom lipid content, which will strengthen the possibility of diatoms being applied in biofuel production. However, many questions still need to be answered about our study. For example, can CRISPRi be used for other genes or diatoms? Why was only one type of fatty acid productivity improved in the mutants? What is the molecular mechanism of the PtECH downregulation that enhances the photosynthesis efficiency of the photosystem II? In this regard, our study is more like throwing a stone to test the waters.

## 5. Conclusions

To sum up, we have developed a CRISPRi technology to inhibit the expression of the enoyl-CoA hydratase gene in *P. tricornutum*. Our findings show that the two knockdown mutants exhibited a reduction in enoyl-CoA hydratase mRNA levels compared to the wild type. However, the knockdown of the enoyl-CoA hydratase gene resulted in increased lipid and chlorophyll content in *P. tricornutum*. This study introduces a new strategy for enhancing lipid content in *P. tricornutum*.

## Data Availability

The datasets generated and/or analyzed during the current study are included in this published article.

## Supplementary Materials

The following supporting information can be downloaded at: https://www.mdpi.com/article/10.3390/cimb46100649/s1, Figure S1: Domain and phylogenetic analysis of ECH proteins; Table S1: Primers used in this study.
