# Supplementary material for: Improving Lipid Content in the Diatom Phaeodactylum tricornutum by the Knockdown of the Enoyl-CoA Hydratase Using CRISPR Interference
Source: Curr Issues Mol Biol. 2024 Sep 28;46(10):10923–33. doi: 10.3390/cimb46100649 (PMC11506698; doi:10.3390/cimb46100649)
Supplement: Supplementary file 1 [file cimb-46-00649-s001.zip › supplementary Table S1.pdf]

Table S1. Primers used in this study

Primers	Sequence (5’-3’)
cas9D10A-F	TACTCGATTGGATTGGCTATCGGCACCAACTCT
cas9D10A-R	AGAGTTGGTGCCGATAGCCAATCCAATCGAGTA
cas9H840A-F	CGATTACGATGTGGACGCCATTGTTCCTCAGTCGT
cas9H840A-R	ACGACTGAGGAACAATGGCGTCCACATCGTAATCG
ptech21-T	TCGATCCACCGCAGGTAACAATGG
ptech21-B	AAACCCATTGTTACCTGCGGTGGA
ptech1487-T	TCGAGATCCACGCCGCCACCGCCT
ptech1487-B	AAACAGGCGGTGGCGGCGTGGATC
ptech-TF	AGCCATCCTTTCTGCCCG
ptech-TR	AGCGGATAACAATTTCACACAGGA
H4-QF	CGTCGTGGTGGTGTGAAAC
H4-QR	TGCTCGGTATAGGTGACGGA
pt55192-QF	CAATGCGTCACAAGAATTACCG
pt55192-QR	CATACCTTCCGTTTGATCCTCG
