# Supplementary figures and images for: Improving Lipid Content in the Diatom Phaeodactylum tricornutum by the Knockdown of the Enoyl-CoA Hydratase Using CRISPR Interference
Source: Curr Issues Mol Biol. 2024 Sep 28;46(10):10923–33. doi: 10.3390/cimb46100649 (PMC11506698; doi:10.3390/cimb46100649)

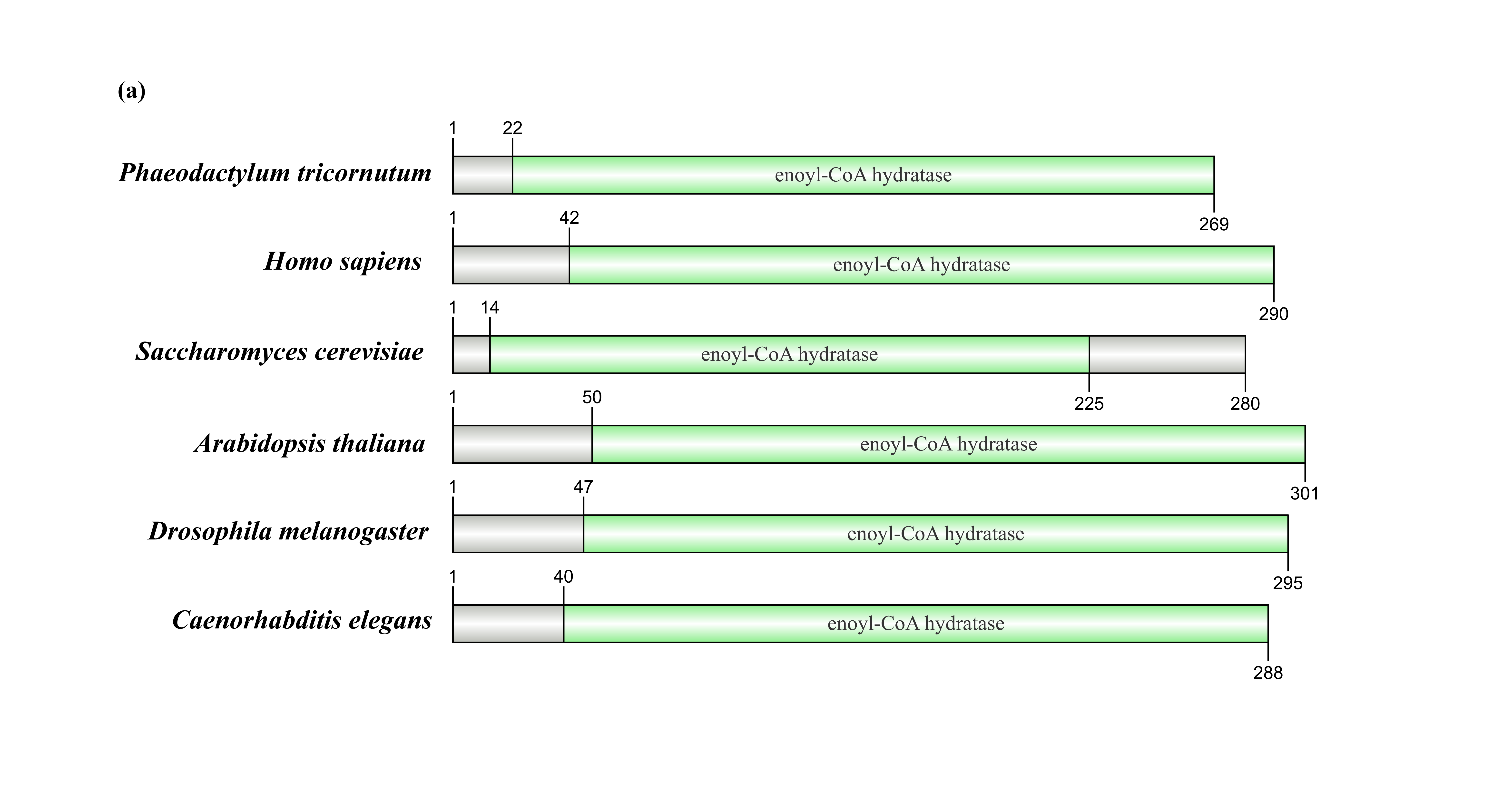

Supplement: Supplementary file 1 [file cimb-46-00649-s001.zip › supplementary figure 1a .tif]

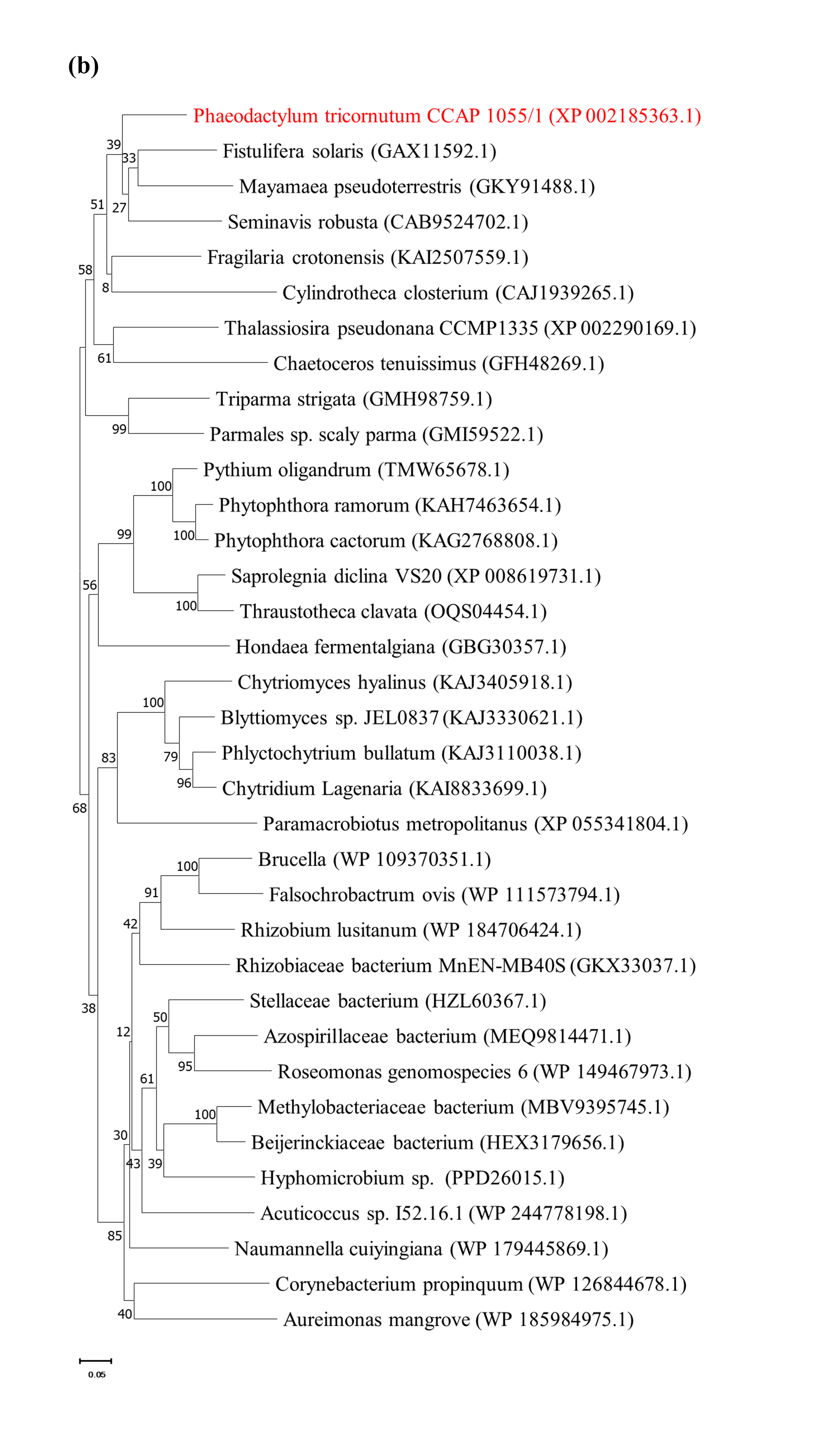

Supplement: Supplementary file 1 [file cimb-46-00649-s001.zip › supplementary figure 1b .tif]
